# A cross-cohort comparison of the prevalence and clinical significance of Alzheimer’s disease biomarkers in people with versus without HIV

**DOI:** 10.1007/s13365-026-01309-7

**Published:** 2026-03-25

**Authors:** Lillian Ham, Olivia Villers, Judith D. Lobo, Tyler R. Bell, Debralee Cookson, Douglas Galasko, Scott L. Letendre, Mark W. Bondi, David J. Moore, Erin E. Sundermann

**Affiliations:** 1 San Diego State University/University of California San Diego Joint Doctoral Program in Clinical Psychology, San Diego, CA USA; 2 HIV Neurobehavioral Research Program, University of California San Diego, 220 Dickinson Street, Suite B, San Diego, CA 92103 USA; 3 University of California San Diego School of Medicine, La Jolla, CA USA; 4 Department of Psychiatry, University of California San Diego, La Jolla, CA USA; 5 Department of Neurosciences, University of California San Diego, La Jolla, CA USA

**Keywords:** Cognition, Dementia, Aging, Amyloid, Tauopathies, Neurocognitive disorders

## Abstract

With over half of people with HIV (PWH) in the U.S. entering older adulthood, identifying markers to distinguish Alzheimer’s disease (AD) and its precursor, amnestic mild cognitive impairment (aMCI), from other forms of neurocognitive impairment among PWH is urgent. We examined how HIV and aMCI status relate to AD CSF biomarkers in adult PWH and people without HIV (PWoH) characterized for aMCI. We included 80 PWH from the National NeuroHIV Tissue Consortium and 80 PWoH from the Wisconsin Registry for Alzheimer’s Prevention. Binary logistic regressions of AD-related CSF biomarker positivity (Aβ_42,_ Aβ_42_/Aβ_40,_ t-tau, p-tau_181_, Aβ_42_/t-tau) were conducted with HIV serostatus, aMCI status, HIV x aMCI interaction, and demographic covariates. Among PWH, we examined how HIV-disease characteristics relate to AD biomarker positivity. No HIV x aMCI interactions were detected. Regardless of aMCI status, having HIV was associated with higher odds of CSF Aβ_42_ (OR = 7.97) and Aβ_42_/Aβ_40_ (OR = 6.06) positivity, suggesting increased cerebral Aβ plaque burden. Regardless of HIV serostatus, having aMCI was associated with higher odds of CSF p-tau_181_ positivity (OR = 3.64). Neither HIV nor aMCI status related to Aβ_42_/t-tau positivity. No HIV-disease characteristics related to AD biomarker positivity. Higher CSF Aβ positivity in PWH versus PWoH, regardless of aMCI status, suggests that HIV disease promotes amyloidosis; however, whether positive CSF biomarkers in PWH raises risk for future cognitive impairment and AD remains to be explored longitudinally. Like PWoH, elevated CSF p-tau_181_ among PWH may indicate increased risk for AD-related memory impairment.

## Introduction

In addition to HIV-associated neurocognitive disorders (HAND) (Heaton et al. 2010), aging people with HIV (PWH) face risk of age-related neurodegenerative conditions such as Alzheimer’s disease (AD) and its precursor, amnestic mild cognitive impairment (aMCI). Whether PWH are at higher risk for AD compared to the general population remains unclear, but some evidence suggests PWH exhibit atypical aging (Pathai et al. 2014). This concern is heightened by AD and HIV sharing biological risk factors (Deeks 2011) (e.g., inflammation, immune senescence), highlighting the need for investigation of AD risk and detection in PWH (Rubin et al. 2019).

Amyloid-β (Aβ) plaques and neurofibrillary tangles composed of hyperphosphorylated-tau (p-tau) aggregates are hallmark pathological features of AD. Aβ plaques typically appear before clinical symptoms while tau pathology increases with the severity of cognitive impairment (Jack et al. 2010), although others have offered that tau pathology may be co-incident with or pre-date significant amyloidopathy (Braak and Del Tredici 2015). Evidence regarding in-vivo cerebrospinal fluid (CSF) AD biomarkers in PWH versus PWoH and their relationship to cognition is inconsistent. Multiple studies have found that CSF Aβ is lower (suggesting greater cerebral Aβ plaque burden) in older PWH and PWH with HAND compared to cognitively unimpaired PWoH (Brew et al. 2005; Clifford et al. 2009; de Almeida et al. 2018, 2020; Krut et al. 2013), though still generally higher than CSF Aβ levels in people with AD (Ances et al. 2012; Cooley et al. 2023; de Almeida et al. 2018). Among PWH, HAND has been associated with reduced CSF Aβ levels (Clifford et al. 2009; Fields et al. 2020; Krut et al. 2013); however, others have found no relationship between cognition and CSF Aβ in PWH (Cooley et al. 2023; Ellis et al. 2022). Regarding tau pathology, p-tau is a more specific AD biomarker, while total tau (t-tau) indicates non-specific axonal injury. CSF p-tau and t-tau elevations found among PWoH with AD are often absent in PWH with HAND (Clifford et al. 2009; de Almeida et al. 2018; Krut et al. 2013); however, increased CSF t-tau (Brew et al. 2005; Ellis et al. 2022; Gisslen et al. 2009) and p-tau (Brew et al. 2005; Cysique et al. 2015) levels have been correlated with greater cognitive impairment among PWH.

Many published studies have methodological limitations, including (1) large-scale mean age differences, often by 20–30 years, between PWH and PWoH; (2) absence of PWoH in earlier stages of AD-related clinical expression such as aMCI; and (3) lack of aMCI profiles in PWH that may be more germane to analyses of AD biomarkers. Studies that compare age-matched PWH and PWoH on CSF AD biomarkers focusing on earlier stages of AD would address these limitations.

Accurately identifying AD-related aMCI among PWH is necessary for intervention and life planning, but is challenging since episodic memory impairment is a defining feature of aMCI and common in HAND (Woods et al. 2009). To identify aMCI cases in PWH, our research group leveraged differences in the memory deficit profile between aMCI and HAND (Sundermann et al. 2021). While HAND is typically characterized by a frontal and subcortical-based memory impairment profile involving impaired memory retrieval (i.e., recall) with intact retention (i.e., recognition) (Woods et al. 2009), aMCI and AD are characterized by a hippocampal-based memory impairment profile of both retrieval and retention. We adapted the Jak/Bondi criteria for aMCI (Bondi et al. 2014; Jak et al. 2009) to improve the ability to distinguish it from HAND in PWH. This approach is supported by a post-mortem study which found that aMCI diagnosis within a year of death was associated with a higher likelihood of Aβ pathology in frontal brain tissue among PWH (Sundermann et al. 2021).

### Study aims and hypotheses

We intentionally leveraged the rich in-life cognitive characterization and AD CSF biomarker data from the Wisconsin Registry for Alzheimer’s Prevention (WRAP) as a comparison cohort to PWH from the National NeuroHIV Tissue Consortium (NNTC). The WRAP is a mid-to-late life cohort enriched with individuals who are predisposed to developing AD. The WRAP sample allows for a better age-matched comparison of PWH versus PWoH on cognition and AD biomarkers as compared to a cohort of PWoH who have AD.

We examined how HIV serostatus, aMCI status, and their combination relates to CSF AD biomarkers of amyloid and tau. We hypothesized independent main effects of HIV and aMCI status on Aβ_42_ and Aβ_42_/Aβ_40_ positivity with higher rates of positivity among PWH versus PWoH and aMCI versus non-aMCI individuals. We additionally hypothesized a main effect of aMCI, but not HIV, status on p-tau_181_, t-tau, and Aβ_42_/t-tau with higher rates of positivity among aMCI versus non-aMCI individuals. Among PWH only, we explored whether AD biomarker positivity was associated with HIV-related disease characteristics.

## Methods

### Participants

#### NNTC cohort

Participants included 80 PWH from the NNTC, a multi-site longitudinal and organ donation study, enrolled at one of four human tissue banks: Galveston, Texas; Los Angeles, California; New York City, New York; and San Diego, California. Participants provided written informed consent to undergo study procedures, which were approved by the Institutional Review Board (IRB) at each medical research center. Further details regarding recruitment and study procedures are published elsewhere (Woods et al. 2004). PWH completed visits between 1999 and 2015 including CSF AD biomarker testing, a comprehensive neurocognitive evaluation, and assessment of medical and psychiatric conditions. Major depressive disorder (MDD) was assessed using the Composite International Diagnostic Interview based on *Diagnostic and Statistical Manual of Mental Illnesses (DSM-IV)* criteria (Organization 1997).

#### WRAP cohort

Data were extracted from the WRAP dataset, a multi-site longitudinal observational study of late middle-aged and older adults, enriched for risk of AD by way of parental history of AD. Parental family history of AD was defined as having at least one biological parent with probable AD based on: (1) NINDS-ADRDA criteria (McKhann et al. 1984), (2) autopsy, or (3) the Dementia Questionnaire (Ellis et al. 1998); see (Johnson et al. 2018) for more detail. Participants provided written informed consent to undergo study procedures approved by the IRB of the University of Wisconsin-Madison. Further details regarding recruitment and study procedures are published elsewhere (Johnson et al. 2018). Participants completed visits between 2001 and 2019 and were assessed for MDD, hypertension, and diabetes via self-report. We received a dataset of 135 PWoH who completed CSF AD biomarker testing and a comprehensive neurocognitive evaluation. From this pool of 135 WRAP participants, we selected the youngest 73 participants and 7 participants who were assigned an aMCI diagnosis to better match our NNTC cohort on age and level of cognitive impairment. The participants were selected blinded to biomarker data.

### CSF AD biomarkers

In the NNTC cohort, all CSF biomarker concentrations were measured using commercially available immunoassays. Levels of p-tau_181_ and t-tau were measured using bead suspension arrays (Luminex, Millipore, Massachusetts), and Aβ_42_ and Aβ_40_ were measured using electrochemiluminescence arrays (Meso Scale Discovery, Maryland; Table 1). In the WRAP cohort, CSF levels of p-tau_181_, t-tau, Aβ_42,_ and Aβ_40_ were performed on a Cobas e601n analyzer (Elecsys) (Van Hulle et al. 2021). Greater pathological burden and increased AD risk was indicated by lower levels of Aβ_42,_ Aβ_42_/Aβ_40,_ and Aβ_42_/t-tau, and higher levels of p-tau_181_ and t-tau.

**Table 1:** Alzheimer’s disease biomarker positivity cutoffs

CSF biomarker	NNTC assay	Cut-off (pg/mL)	WRAP assay	Cut-off (pg/mL)
Aβ_42_	MSD	≤ 506.00^a^	Elecsys	≤ 939.15^d^
Aβ_42_/Aβ_40_	MSD	≤ 0.08^a^	Elecsys	≤ 0.05^e^
t-tau	MSD	≥ 470.00^b^	Elecsys	≥ 293.40^e^
p-tau_181_	Luminex	≥ 64.54^c^	Elecsys	≥ 24.80^e^
Aβ_42_/t-tau	MSD/MSD	≤ 1.39^a^	Elecsys	≥ 0.26^d†^

Note. *NNTC* National NeuroHIV Tissue Consortium, *WRAP *Wisconsin Registry for Alzheimer’s Prevention, *MSD* Meso Scale Discovery. ^†^Cut-off based on t-tau/Aβ_42_ but labeled as “Aβ_42_/t-tau” to be consistent across cohorts. ^a^(Janelidze et al. 2017), ^b^(Pan et al. 2015), ^c^(Hansen et al. 2021), ^d^(Dakterzada et al. 2021), ^e^(Van Hulle et al. 2021)

Due to differences in assay platforms used to derive AD biomarkers in the NNTC versus the WRAP cohort, AD biomarker levels were dichotomized for amyloid positivity based on predefined cutoff values per assay type to allow for standardized cross-cohort comparisons (Table 1). For descriptive purposes, we log-transformed biomarker concentration levels to improve normality and report medians and IQR by cohort (Table 2).

**Table 2:** Sample characteristics by cohort

	Total	NNTC (PWH)	WRAP (PWoH)	
	*N* = 160	*n* = 80	*n* = 80	*p*
*Demographics*				
Age (yrs), M (SD)	52.4 (10.2)	45.7 (9.4)	59.1 (5.7)	**< 0.01** ^**a**^
Race, n (%)				
American Indian/NA	2 (1.3)	2 (2.5)	0 (0.0)	**< 0.01** ^**b**^
Asian	1 (0.6)	1 (1.3)	0 (0.0)	
Black	26 (16.3)	26 (32.5)	0 (0.0)	
White	130 (81.3)	50 (62.5)	80 (100.0)	
Other	1 (0.6)	1 (1.3)	0 (0.0)	
Education (yrs), M (SD)	14.1 (3.1)	12.2 (2.8)	15.9 (2.2)	**< 0.01**
Male, n (%)	90 (56.3)	63 (78.9)	27 (33.8)	**< 0.01**
*Comorbidities*				
Hypertension^c^, n (%)	17 (13.5)	6 (12.8)	11 (13.9)	0.85
Diabetes^d^, n (%)	4 (3.2)	4 (8.5)	0 (0.0)	**< 0.01**
Lifetime depression^e^, n (%)	55 (35.9)	46 (63.0)	9 (11.3)	**< 0.01**
aMCI+, n (%)	52 (32.5)	34 (42.5)	18 (22.5)	**< 0.01**
*HIV disease characteristics*				
HAND^f^, n (%) Other NCI	--	28 (43.8) 18 (28.1)	--	--
HIV duration (yrs)^g^, M (SD)	--	13.9 (6.2)	--	--
Nadir CD4 (c/µL)^h^, mdn [IQR]	--	18.5 [3.0-67.5]	--	--
Current CD4 (c/µL)^i^, mdn [IQR]	--	76.0 [9.5–226.0]	--	--
Plasma viral load^j^ <200 (copies/mL), n (%)	--	27 (36.0)	--	--
CSF viral load^k^ <200 (copies/mL), n (%)	--	46 (67.6)	--	--
On ART^l^, n (%)	--	42 (60.9)	--	--
*AD biomarkers*,* pg/mL*				
Log Aβ_42_	--	5.6 [5.2-6.0]	6.8 [6.5–7.2]	--
Log + 1 Aβ_42_/Aβ_40_	--	0.1 [0.1–0.1]	0.1 [0.1–0.1]	--
Log p-tau_181_	--	3.7 [3.3-4.0]	2.9 [2.6–3.1]	--
Log t-tau	--	4.3 [3.4–4.8]	5.3 [5.1–5.5]	--
Log + 1 Aβ_42_/t-tau	--	1.7 [1.3–2.2]	1.8 [1.6-2.0]	--

Note. *NNTC *National NeuroHIV Tissue Consortium, *WRAP*  Wisconsin Registry for Alzheimer’s Prevention, *yrs* years, *NA *Native American, *aMCI *+ amnestic mild cognitive impairment present, *aMCI*- aMCI absent, *HAND *HIV-associated neurocognitive disorder, *NCI *neurocognitive impairment, *ART *antiretroviral therapy, *M *mean, *SD *standard deviation, *mdn *median, *IQR *interquartile range. ^a^Welch’s t-test for unequal variances; ^b^White vs. non-White; ^c^NNTC *n* = 47, WRAP *n* = 79; ^d^NNTC *n* = 47, WRAP *n* = 80; ^e^NNTC *n* = 73, WRAP *n* = 80; ^f^NNTC *n* = 64; ^g^NNTC *n* = 75; ^h^NNTC *n* = 62; ^i^NNTC *n* = 73; ^j^NNTC *n* = 75; ^k^NNTC *n* = 68; ^l^NNTC *n* = 69. *p*-values < 0.05 are bolded and denote statistical significance

### Neurocognitive evaluation and aMCI classification

Both NNTC and WRAP participants completed a standardized neurocognitive battery including domains of attention/working memory, processing speed, executive function, learning, memory, verbal fluency, and motor function. For both cohorts, visual memory was measured via the Brief Visuospatial Memory Test-Revised (BVMT-R). Verbal memory was measured via similar list verbal learning tests: the Hopkins Verbal Learning Test-Revised (HVLT-R) for the NNTC cohort, and the Rey Auditory Verbal Learning Test (AVLT) for the WRAP cohort. Learning, delayed recall, and recognition raw scores were converted to T-scores and demographically adjusted for age, education, sex, and race for the BVMT-R and HVLT-R (Heaton 2004), and age, education, and sex for the AVLT (Stricker et al. 2021).

To assign aMCI diagnoses in both cohorts, an adapted version of the Jak/Bondi neuropsychological MCI criteria was applied (Jak et al. 2009; Sundermann et al. 2021). An aMCI diagnosis required two of six possible impaired (> 1 SD below the normative mean) memory T-scores among BVMT-R and HVLT-R or AVLT learning, recall, or recognition scores with at least one impaired score being recognition. For descriptive purposes, PWH were further classified by HAND status via Frascati criteria (Antinori et al. 2007) or with neurocognitive impairment due to other causes.

### Statistical analyses

Sample characteristics were compared between PWH (NNTC) vs. PWoH (WRAP) using independent samples t-tests or Welch’s t-tests for unequal variances for continuous outcomes and chi-square tests of independence or Fisher’s exact for categorical outcomes. Sample characteristics and positivity rates for each AD biomarker were also compared among HIV serostatus by aMCI status subgroups (PWoH/aMCI-, PWoH/aMCI+, PWH/aMCI-, PWH/aMCI+) using one-way ANOVAs or Welch’s ANOVAs and chi-square tests of independence or Fisher’s exact tests. HIV-disease characteristics were compared between PWH groups (PWH/aMCI + vs. PWH/aMCI-) using independent samples t-tests or non-parametric Wilcoxon two-sample tests for variables with non-normal distributions. Significant omnibus tests were followed up by pairwise comparisons corrected for multiple comparisons using the Benjamini-Hochberg method.

For Aim 1, the following predictors were included in binary logistic regressions predicting odds of AD biomarker positivity: HIV serostatus, aMCI status, HIV x aMCI interaction, and demographics (age, sex, White race, years of education). Non-significant interaction effects were removed from the final model to interpret main effects. For Aim 2, the following HIV-disease characteristics were included in binary logistic regressions predicting odds of AD biomarker positivity among PWH: years of HIV infection, nadir CD4 count (log_10_+1 transformed to improve normality), current CD4 count (log_10_+1 transformed to improve normality), plasma HIV viral load (< 200 copies/mL vs. ≥ 200 copies/mL), and ART status (on vs. off). Due to substantial variability in missing data (Table 2), models were conducted separately for each HIV-disease characteristic controlling for demographics. Other considered covariates included lifetime MDD diagnosis, hypertension, and diabetes (Table 2). Significant missing data on hypertension and diabetes in the NNTC cohort (41.3%) precluded inclusion of these covariates; sensitivity analyses were conducted including these covariates to assess change in results. Two-tailed tests with a significance level of 0.05 were conducted in JMP Pro version 18.0.1 and IBM SPSS version 29.0.2.0.

## Results

### Characterization of cohorts and HIV/aMCI subgroups

In total, 67.9% of the WRAP cohort (PWoH) reported having a family history of AD. Compared to the NNTC cohort (PWH), the WRAP cohort was on average 15 years older, composed of only White participants, and had greater years of education (Table 2). The NNTC participants were more likely to be male and have diabetes, lifetime MDD, and aMCI than WRAP participants. Among PWH, 43.8% had HAND and 53.6% of PWH with HAND were also classified with aMCI+.

**Fig. 1:**
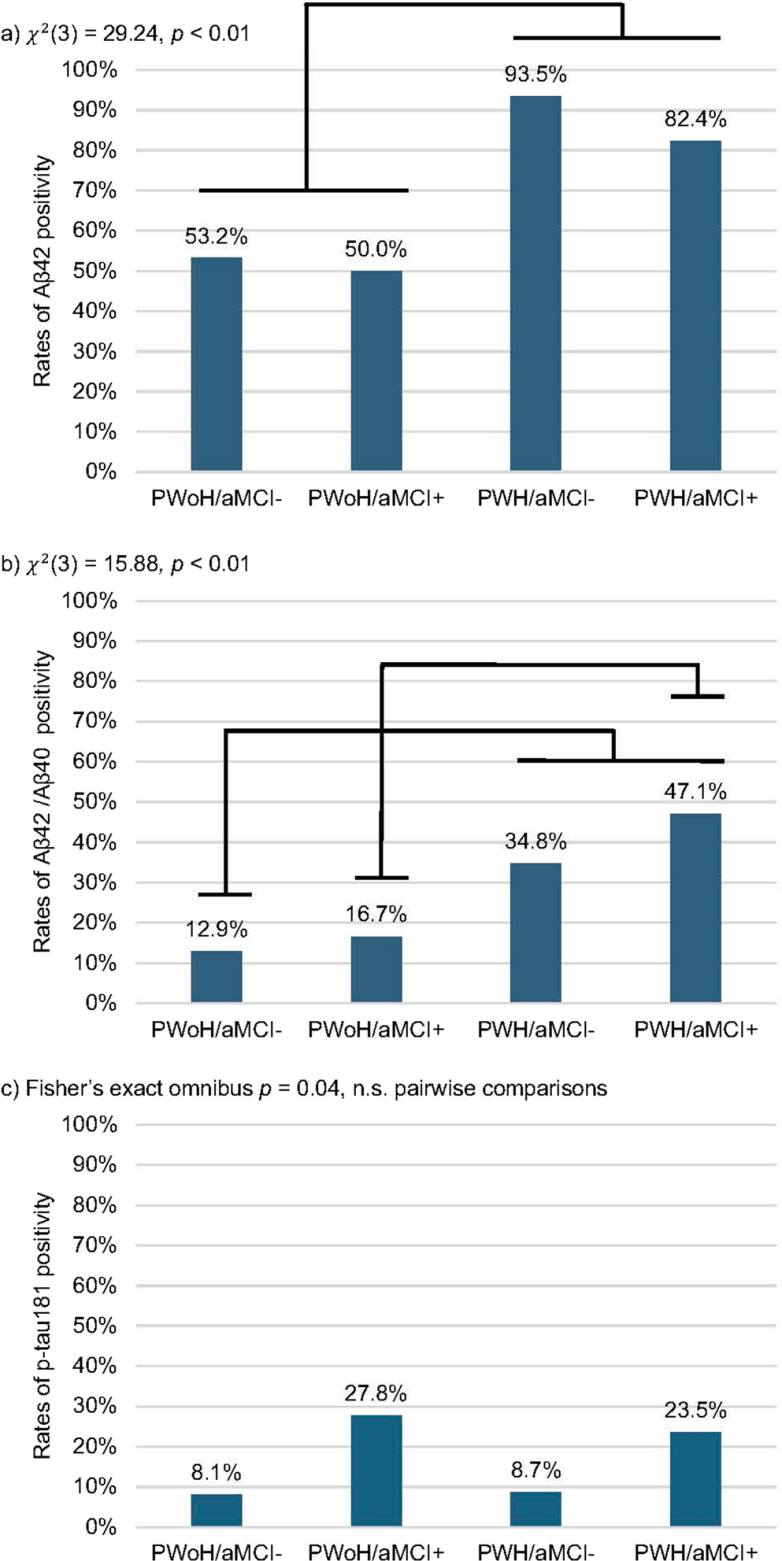
Rates of Alzheimer's disease biomarker positivity across HIV/aMCI subgroup unadjusted for covariates. Omnibus test statistics and p-values are reported. Black bars represent statistically significant pairwise comparisons after Benjamini-Hochberg correction. Sample sizes: PWoH/aMCI- = 62, PWoH/aMCI+ = 18, PWH/aMCI- = 46, PWH/aMCI+ = 34. PWH = people with HIV, PWoH = people without HIV, aMCI = amnestic mild cognitive impairment

Rates of AD biomarker positivity for the total sample were as follows: 70.6% (*n* = 113) for Aβ_42_, 26.9% (*n* = 43) for Aβ_42_/Aβ_40,_ 13.8% (*n* = 22) for p-tau_181_, 6.9% (*n* = 11) for t-tau, and 16.3% (*n* = 26) for Aβ_42_/t-tau. The total sample was classified into four subgroups: 38.8% (*n* = 62) as PWoH/aMCI-, 1.3% (*n* = 18) as PWoH/aMCI+, 28.8% (*n* = 46) as PWH/aMCI-, and 21.3% (*n* = 34) as PWH/aMCI+. Sample characteristics including rates of AD biomarker positivity by subgroup are provided in Table 3. There were only two PWH (one in the aMCI+ group and one in the aMCI- group) that were classified as t-tau positive; therefore, analyses by PWH/aMCI subgroup for t-tau positivity were not conducted due to insufficient statistical power. The PWH/aMCI+ group was mostly male whereas the PWoH/aMCI- group was mostly female (Table 3). PWH/aMCI- were more likely to have diabetes than PWoH/aMCI-. PWH/aMCI+ were more likely to have HAND than PWH/aMCI-; however, HIV disease characteristics did not differ between PWH groups. PWH/aMCI subgroups differed in rates of Aβ_42_, Aβ_42_/Aβ_40_, and p-tau_181_ positivity, but not in rates of Aβ_42_/t-tau positivity (Fig. 1). Regardless of aMCI status, PWH had higher rates of Aβ_42_ and Aβ_42_/Aβ_40_ positivity than PWoH. Although aMCI- groups had lower rates of p-tau_181_ positivity than aMCI+ groups regardless of HIV serostatus, no pairwise comparisons remained significant after adjusting for multiple comparisons.

**Table 3:** Sample characteristics by HIV/aMCI subgroup

	HIV/aMCI subgroup					
	1. PWoH/aMCI-	2. PWoH/aMCI+	3. PWH/aMCI-	4. PWH/aMCI+		
	*n* = 62	*n* = 18	*n* = 46	*n* = 34	Omnibus *p*	Pairwise comparisons
*Demographics*						
Age (yrs), M (SD)	58.3 (4.5)	62.0 (8.2)	45.2 (9.8)	46.4 (8.9)	**< 0.01** ^**a**^	2, 1 > 4, 3
Race, n (%)					**< 0.01** ^**b**^	1, 2 > 3, 4
American Indian/NA	0 (0.0)	0 (0.0)	0 (0.0)	2 (5.9)		
Asian	0 (0.0)	0 (0.0)	1 (2.2)	0 (0.0)		
Black	0 (0.0)	0 (0.0)	13 (28.3)	13 (38.2)		
White	62 (100.0)	18 (100.0)	31 (67.4)	19 (55.9)		
Other	0 (0.0)	0 (0.0)	1 (2.2)	0 (0.0)		
Education (yrs), M (SD)	15.8 (2.2)	16.4 (2.3)	12.3 (2.9)	12.2 (2.7)	**< 0.01**	2, 1 > 3, 4
Male, n (%)	17 (27.4)	10 (55.6)	32 (69.6)	31 (91.2)	**< 0.01**	4 > 3, 2 > 1
*Comorbidities*						
Hypertension, n (%)	7 (11.3)	4 (23.5)	2 (8.0)	4 (18.2)	0.41^c^	--
Diabetes, n (%)	0 (0.0)	0 (0.0)	3 (12.0)	1 (4.6)	**0.03** ^**c**^	3 > 1
LT depression, n (%)	7 (11.3)	2 (11.1)	27 (62.8)	19 (63.3)	**< 0.01**	4, 3 > 1, 2
*HIV disease characteristics* ^*d*^						
HAND^d^, n (%) Other NCI	--	--	13 (36.1) 8 (22.2)	15 (53.6) 10 (35.7)	**0.02**	--
HIV duration (yrs), M (SD)	--	--	14.0 (6.3)	13.7 (6.3)	0.82	--
Nadir CD4 (c/µL), mdn [IQR]	--	--	11.0 [3.0–64.0]	20.0 [4.0–80.0]	0.56^e^	--
Current CD4 (c/µL), mdn [IQR]	--	--	63.0 [8.5–252.0]	81.0 [17.0–219.0.0.0]	0.76^e^	--
Plasma viral load < 200 (copies/mL), n (%)	--	--	16 (38.1)	11 (33.3)	0.67	--
On ART, n (%)	--	--	28 (70.0)	14 (48.3)	0.07	--
*AD biomarker positivity*						
Aβ_42,_ n (%)	33 (53.2)	9 (50.0)	43 (93.5)	28 (82.4)	**< 0.01**	3, 4 > 1, 2
Aβ_42_/Aβ_40,_ n (%)	8 (12.9)	3 (16.7)	16 (34.8)	16 (47.1)	**< 0.01**	4 > 2; 4, 3 > 1
p-tau_181_, n (%)	5 (8.1)	5 (27.8)	4 (8.7)	8 (23.5)	**0.04**	n.s.
t-tau, n (%)	4 (6.5)	5 (27.8)	1 (2.2)	1 (2.9)	--^f^	--
Aβ_42_/t-tau, n (%)	15 (24.2)	3 (16.7)	4 (8.7)	4 (11.8)	0.16	--

Note. ^a^Welch’s ANOVA for unequal variances, ^b^White vs. non-White, ^c^Fisher’s exact test for expected cell counts < 5, ^d^subgroups 3 vs. 4 only, ^e^Wilcoxon two-sample test. ^f^Fisher’s exact test not conducted as only 2 PWH showed t-tau positivity. *PWH* people with HIV, *PWoH* people without HIV, *yrs* years, *NA* Native American, *LT* lifetime, *HAND* HIV-associated neurocognitive disorder, *NCI* neurocognitive impairment, *ART* antiretroviral therapy, *AD* Alzheimer’s disease, *M* mean, *SD* standard deviation, *mdn* median, *IQR* interquartile range. *p*-values < 0.05 are bolded and denote statistical significance

### Main effects of HIV and aMCI on AD biomarker positivity

HIV serostatus was excluded as a predictor in models with t-tau as only 2 PWH had t-tau positivity (Table 4). There were no significant HIV x aMCI interaction effects; thus, these terms were removed from all models. Controlling for aMCI status and demographic variables, having HIV was associated with more than 6 times higher odds of Aβ_42_ (OR = 7.97, 95% CI [1.85, 34.36], *p* < 0.01) and Aβ_42_/Aβ_40_ positivity (OR = 6.06, 95% CI [1.66, 22.13], *p* = 0.01). Controlling for HIV serostatus and demographic variables, having aMCI was associated with 3.64 times higher odds of p-tau_181_ positivity (OR = 3.64, 95% CI [1.33, 9.97], *p* = 0.01). In sensitivity analyses restricted to samples including lifetime MDD, hypertension, and diabetes, findings were unchanged.

**Table 4:** Binary logistic regression of HIV and aMCI status predicting odds of Alzheimer’s disease biomarker positivity

*N* = 160	Aβ_42_			Aβ_42_/Aβ_40_			*p*-tau_181_			t-tau			Aβ_42_/t-tau		
*Predictor*	*OR*	*95% CI*	*p*	*OR*	*95% CI*	*p*	*OR*	*95% CI*	*p*	*OR*	*95% CI*	*p*	*OR*	*95% CI*	*p*
Intercept	2.37	--	0.66	0.01	--	0.01	0.00	--	< 0.01	0.00	--	< 0.01	0.00	--	0.01
PWH (ref. PWoH)	7.97	1.85–34.36	**< 0.01**	6.06	1.66–22.13	**0.01**	4.13	0.79–21.58	0.09	--	--	--	0.94	0.19–4.76	0.95
aMCI+ (ref. aMCI-)	0.65	0.27–1.58	0.34	1.44	0.65–3.21	0.37	3.64	1.33–9.97	**0.01**	3.27	0.79–13.59	0.10	0.75	0.27–2.11	0.59
Age (yrs)	0.99	0.94–1.05	0.84	1.02	0.97–1.07	0.52	1.06	0.99–1.13	0.10	1.09	1.00-1.20	0.06	1.06	0.99–1.14	0.08
Female (ref. male)	0.92	0.39–2.17	0.85	1.20	0.48–3.01	0.70	0.77	0.24–2.53	0.67	0.61	0.15–2.60	0.51	0.89	0.33–2.43	0.82
White (ref. non-White)	0.76	0.18–3.29	0.72	1.23	0.45–3.38	0.69	0.56	0.13–2.48	0.45	5.65	0.56–56.89	0.14	1.34	0.27–6.60	0.72
Education (yrs)	0.98	0.83–1.16	0.83	1.10	0.94–1.29	0.22	1.12	0.92–1.37	0.26	1.44	1.02–2.03	**0.04**	1.07	0.88–1.29	0.51
*Overall model fit*	${\chi}^{2}$(6) = 28.33, *p* < 0.01, Nagelkerke R^2^ = 0.23			${\chi}^{2}$(6) = 19.11, *p* < 0.01, Nagelkerke R^2^ = 0.16			${\chi}^{2}$(6) = 13.52, *p* = 0.04, Nagelkerke R^2^ = 0.15			${\chi}^{2}$(5) = 17.22, *p* < 0.01, Nagelkerke R^2^ = 0.26			${\chi}^{2}$(6) = 9.76, *p* = 0.14, Nagelkerke R^2^ = 0.10		

Note. *Ref*. reference group, *PWH* people with HIV, *PWoH* people without HIV, *aMCI* + amnestic mild cognitive impairment present, *aMCI*- aMCI absent, *yrs* years, *OR* odds ratio, *CI* confidence interval. *p*-values < 0.05 are bolded and denote statistical significance

### Main effects of HIV-related disease characteristics on AD biomarker positivity

Models predicting t-tau positivity were not conducted as only 2 PWH exhibited abnormal t-tau levels (Table 5). Overall, no HIV-disease related characteristics were significantly associated with Aβ_42,_ Aβ_42_/Aβ_40,_ and p-tau_181_. As these non-significant values were likely influenced by low statistical power, we report effect sizes in Table 5. In sensitivity analyses restricted to samples including lifetime MDD, hypertension, and diabetes, findings were unchanged.

**Table 5:** Binary logistic regressions of HIV-related disease characteristics predicting odds of Alzheimer’s disease biomarker positivity

Predictor	Aβ_42_			Aβ_42_/Aβ_40_			*p*-tau_181_			Aβ_42_/t-tau^a^		
	OR	95% CI	*p*	OR	95% CI	*p*	OR	95% CI	*p*	OR	95% CI	*p*
HIV duration (yrs)	0.99	0.87–1.12	0.84	0.95	0.87–1.03	0.21	1.04	0.94–1.16	0.45	0.98	0.87–1.10	0.72
Nadir CD4 (c/µL)	1.15	0.25–5.39	0.86	1.46	0.62–3.43	0.38	1.56	0.46–5.27	0.48	0.79	0.22–2.89	0.72
Current CD4 (c/µL)	1.01	0.23–4.39	0.99	1.15	0.51–2.59	0.74	2.04	0.52–7.93	0.31	1.72	0.42–7.10	0.45
Plasma VL < 200 c/mL (ref. ≥ 200)	0.43	0.08–2.43	0.34	0.69	0.22–2.16	0.52	2.20	0.51–9.53	0.29	3.12	0.54–18.15	0.21
On ART (ref. off ART)	1.58	0.29–8.63	0.60	1.42	0.45–4.48	0.55	0.74	0.17–3.24	0.69	0.55	0.10–3.19	0.51

Note. Separate models were conducted for each predictor. Each model controlled for age, sex, White race, and years of education (not shown). ^a^Sex was not included in models with ART status predicting Aβ_42_/t-tau as no women with ART data exhibited Aβ_42_/t-tau positivity. *Ref*. reference group, *yrs* years, *VL* viral load, *ART* antiretroviral therapy, *OR* odds ratio, *CI* confidence interval. *p*-values < 0.05 are bolded and denote statistical significance

## Discussion

Our first hypothesis was partially supported in that having HIV, but not aMCI, related to more than 6 times higher odds of Aβ_42_ and Aβ_42_/Aβ_40_ positivity, though confidence intervals were large given the small sample sizes. In partial support of our second hypothesis, aMCI classification related to more than 3 times higher odds of p-tau_181_, but not Aβ_42_/t-tau positivity. In exploratory analyses, no HIV-related disease characteristics significantly related to rates of Aβ_42_, Aβ_42_/Aβ_40_, p-tau_181_, and Aβ_42_/t-tau positivity.

Across analyses, rates of Aβ_42_ and Aβ_42_/Aβ_40_ positivity were consistently higher among PWH, regardless of aMCI status. These findings are consistent with prior work reporting lower CSF Aβ_42_ levels (suggesting cerebral Aβ plaque burden) in PWH versus PWoH (Brew et al. 2005; Clifford et al. 2009; de Almeida et al. 2018, 2020; Krut et al. 2013). Furthermore, the lack of differences in Aβ positivity by aMCI status in PWH is in line with studies finding no association between CSF Aβ levels and cognition in PWH (Cooley et al. 2023; Ellis et al. 2022). Our finding of higher amyloid positivity in PWH is noteworthy considering their mean age was 15 years younger than PWoH and older age is strongly associated with greater Aβ pathology. A greater burden of cerebral Aβ plaques in younger PWH versus older PWoH may contribute to evidence for atypical brain aging (Umlauf et al. 2019), which may be associated with HIV-related disease mechanisms (Ortega and Ances 2014) or comorbidities (e.g., cerebrovascular disease, opportunistic infections) (Gisslen et al. 2009) known to reduce Aβ clearance, increase plaque development, and are more prevalent in this population. Alternatively, Aβ may play an initial protective antimicrobial role against infection, with chronic activation of innate immunity eventually leading to inflammation and neurodegeneration as seen in AD (Moir et al. 2018).

CSF Aβ biomarkers did not relate to aMCI status, suggesting that CSF Aβ levels are not reflective of current memory deficits in PWH; however, it remains unclear as to whether greater CSF Aβ positivity reflects greater risk of AD as cortical plaques are known to appear a decade or more before clinical symptoms emerge in the AD trajectory (Jack et al. 2010). Longitudinal studies among mid-to-late life PWH that track changes in Aβ in relation to incident aMCI/AD are needed to better understand the clinical significance of greater Aβ burden in PWH. Rates of p-tau_181_ positivity were higher among people with aMCI, regardless of HIV serostatus, suggesting that p-tau_181_ may be a reliable marker of an AD trajectory among PWH.

Taken together, the absence of associations between amyloid and cognition, and the presence of such associations with p-tau_181_, are consistent with prior literature observing that amyloid has negligible to weak associations with cognition in contrast to the robust correlations to cognition with tau pathology (Tanner et al. 2022). That p-tau_181_ has been largely found unrelated to cognition among PWH in prior work may be partially explained by the fact that past studies have examined impairment across multiple cognitive domains (e.g., HAND) rather than a more focused anterograde memory deficit profile characteristic of AD. The lack of a relationship between aMCI status and t-tau supports the general literature that this marker of overall neuronal injury is not AD-specific.

There were no significant associations between AD biomarker positivity and HIV-related disease characteristics. Though the lack of statistical significance across these relationships could be influenced by our limited sample size and reduced statistical power, others have similarly not found robust associations (Cooley et al. 2023; Trunfio et al. 2022). Moreover, effect sizes for most associations were small to medium. Future work is needed to examine the differential relationships between HIV-disease factors across various AD biomarkers.

The current study has several strengths. To our knowledge, no study has compared cognitively characterized PWH and individuals at higher risk for AD (who are more age comparable) to understand differences in AD CSF biomarkers. Prior work has primarily compared AD biomarkers between PWH with HAND, age-matched cognitively normal PWoH or PWH, and individuals with AD who are typically 3–4 decades older than the PWH cohort in this sample. By leveraging the WRAP cohort of late middle-aged and older PWoH at risk for AD, we provided a more age comparable comparison for PWH and compared AD biomarkers by HIV serostatus in prodromal stages of the AD trajectory. Investigating this prodromal stage of aMCI is likely more relevant than AD given that PWH cohorts are primarily comprised of mid-life adults. By using established, assay-specific thresholds to define AD biomarker positivity, we mitigated the influence of platform variability and allowed for valid cross-cohort comparison of AD-related pathology. Referencing published thresholds also contributes towards harmonization of highly heterogeneous biomarker studies in the field. Conducting cross-cohort comparisons maximizes the use of large datasets and avoids siloing of research findings across disease specific disciplines (e.g., researchers working on AD or HIV in isolation). Specifically, the comprehensive and similar memory assessments in both cohorts allowed us to compare AD biomarkers by both HIV and aMCI status.

There were several limitations. Despite our best effort to create age comparability across cohorts, PWoH were still older on average by 15 years than PWH. To minimize demographic differences, we statistically controlled for age, sex, race, and education in models predicting biomarkers, which was a relative improvement compared to past studies. In final binary logistic regressions adjusting for demographics, only education emerged as a significant covariate in a model predicting t-tau. The measurement of AD biomarkers via different assay platforms in WRAP versus NNTC forced us to compare dichotomous versus continuous quantitative biomarker outcomes. While our use of published CSF AD biomarker cut-offs by assay type mitigated these challenges, they may not fully correct for differences in sensitivity and specificity. Without evidence by amyloid PET or autopsy, it is difficult to be certain that lower CSF Aβ_42_ levels among older PWH reflects greater cerebral Aβ plaque burden (Ances et al. 2010). Some subgroup analyses, particularly those involving biomarker positivity classifications, included small numbers of participants, which may reduce the stability and generalizability of those specific estimates. Missing comorbidity data limited our ability to account for these covariates, though sensitivity analyses did not change our primary findings. Family history of AD or other neurodegenerative conditions was not collected via a standardized or reliable method in the NNTC cohort. The WRAP cohort was comprised entirely of White participants, limiting the generalizability of our findings particularly as Black/African American and Hispanic individuals in the U.S. exhibit a disproportionately high prevalence of AD (Matthews et al. 2019). These findings provide preliminary, hypothesis-generating evidence that lays the groundwork for future studies with larger cohorts of individuals with AD biomarker positivity to examine how AD biomarkers relate to HIV serostatus and multi-domain cognition in PWH versus PWoH.

In conclusion, higher CSF Aβ positivity among adult PWH versus PWoH suggests that HIV disease promotes amyloidosis. Further work is needed to examine whether positive AD biomarkers in PWH increases risk for future cognitive impairment and possibly AD. Like PWoH, aMCI classification was associated with higher CSF p-tau_181_ levels among PWH, suggesting that this biomarker may be a reliable indicator of increased AD risk in the context of HIV.

## Data Availability

To protect the privacy of participants, including information on HIV serostatus, data are not made publicly available. NNTC data may be requested (https://nntc.org/) and public reports reflecting the central database are viewable online. WRAP data may similarly be requested (https://wrap.wisc.edu/).
